# Ventrolateral Origin of Each Cycle of Rhythmic Activity Generated by the Spinal Cord of the Chick Embryo

**DOI:** 10.1371/journal.pone.0000417

**Published:** 2007-05-02

**Authors:** Yoshiyasu Arai, George Z. Mentis, Jiang-young Wu, Michael J. O'Donovan

**Affiliations:** 1 Laboratory of Neural Control, Section on Developmental Neurobiology, National Institute of Neurological Disorders and Stroke (NINDS), National Institutes of Health (NIH), Bethesda, Maryland, United States of America; 2 Department of Physiology and Biophysics, Georgetown University, Washington, D. C., United States of America; Medical College of Georgia, United States of America

## Abstract

**Background:**

The mechanisms responsible for generating rhythmic motor activity in the developing spinal cord of the chick embryo are poorly understood. Here we investigate whether the activity of motoneurons occurs before other neuronal populations at the beginning of each cycle of rhythmic discharge.

**Methodology/Principal Findings:**

The spatiotemporal organization of neural activity in transverse slices of the lumbosacral cord of the chick embryo (E8-E11) was investigated using intrinsic and voltage-sensitive dye (VSD) imaging. VSD signals accompanying episodes of activity comprised a rhythmic decrease in light transmission that corresponded to each cycle of electrical activity recorded from the ipsilateral ventral root. The rhythmic signals were widely synchronized across the cord face, and the largest signal amplitude was in the ventrolateral region where motoneurons are located. In unstained slices we recorded two classes of intrinsic signal. In the first, an episode of rhythmic activity was accompanied by a slow decrease in light transmission that peaked in the dorsal horn and decayed dorsoventrally. Superimposed on this signal was a much smaller rhythmic increase in transmission that was coincident with each cycle of discharge and whose amplitude and spatial distribution was similar to that of the VSD signals. At the onset of a spontaneously occurring episode and each subsequent cycle, both the intrinsic and VSD signals originated within the lateral motor column and spread medially and then dorsally. By contrast, following a dorsal root stimulus, the optical signals originated within the dorsal horn and traveled ventrally to reach the lateral motor column.

**Conclusions/Significance:**

These findings suggest that motoneuron activity contributes to the initiation of each cycle of rhythmic activity, and that motoneuron and/or R-interneuron synapses are a plausible site for the activity-dependent synaptic depression that modeling studies have identified as a critical mechanism for cycling within an episode.

## Introduction

The spontaneous activity exhibited by developing networks is believed to be critical for various aspects of neural and network development [1]–[5]. For this reason, it is important to understand the mechanisms regulating its initiation and maintenance. In the spinal cord of the chick embryo, neural networks are activated in episodes of rhythmic discharge separated by longer periods of quiescence [6]–[7]. We have proposed that episodes of activity are regulated by a slow form of activity-dependent network depression that operates over several minutes [8]. One of the mechanisms contributing to this is a redistribution of chloride ions that occurs during the episode [9]–[11]. Much less is known, however, about the rhythmicity that occurs within an episode.

Work in various species has identified several mechanisms that have been proposed to underlie rhythmic activity. In the spinal cord or spinal cord cultures, these include reciprocal inhibition between antagonistic centers, short term synaptic depression, rebound excitation and oscillatory activity induced by NMDA [for reviews see 12]–[14]. In the chick spinal cord, modeling studies have suggested that cycling within an episode is regulated by a fast form of synaptic depression whose time constant is longer than that of the recurrent excitation [8]. Little is known, however, about the source of excitation responsible for each cycle of activity and the critical synapses subject to this hypothesized fast synaptic depression. In studies of locomotor-like rhythmic activity in the mammalian cord, it is generally assumed that the excitatory drive responsible for the rhythmic activation of motoneurons is derived from ventrally located spinal interneurons and that motoneurons are output elements of the cord that are not causally involved in rhythmogenesis [14]. However, in the developing frog spinal cord, it is known that motoneurons contribute to the rhythmic synaptic drive responsible for swimming [15].

The idea that activity in motoneurons might also be important for the genesis of rhythmic activity in the chick was suggested by earlier studies showing that activity in motoneurons and their intraspinal synaptic targets (R-interneurons) precedes that of neurons in other regions of the cord at the beginning of a spontaneous episode [16]–[18]. Unfortunately, in the previous work, it was not possible to investigate the origin of activity in each cycle because calcium signals are relatively slow compared to electrical activity and because video-rate imaging integrates the optical signals within a single frame. For this reason, in the present work we used high speed multi-site optical recording from an *en bloc* slice preparation of the chick embryo spinal cord (E8 to E11) labeled with a voltage sensitive dye to establish the spatial origin of activity at the onset of each cycle. Although several previous studies have used voltage-sensitive dyes to analyze activity in the developing chick spinal cord [19]–[23] none have focused on the spatiotemporal organization of activity during episodes of rhythmic bursting. Some of this work has appeared in abstract form [24].

## Materials and Methods

### Preparations

Chick embryos were removed from the egg at embryonic day 8(E8) to 11(E11) and staged according to the criteria of Hamburger and Hamilton [25]. Embryos were decapitated, and the spinal cords isolated in recirculating cold (12–15°C) Tyrode's solution (in mM: NaCl 139, KCl 2.9, NaHCO_3_ 17, glucose 2.2, CaCl_2_ 3, and MgCl_2_ 1) equilibrated with 95%O_2_/5%CO_2_ [26]. Transverse slices (lumbosacral segment 2) were cut from the isolated cord using a scalpel blade with care taken to preserve the dorsal and ventral roots intact. The thickness of each slice was about 1mm, corresponding to about one segment of the cord. The perfusion solution was slowly brought to room temperature (∼21–22°C), and then the cord was transferred to a recording chamber where it was pinned to the bottom using tungsten wires. The cord was left there for at least 2hr before raising the temperature to ∼27°C for the rest of the experiment.

### Voltage-sensitive dye staining

Slices were stained by incubating them for 20min in Tyrode's solution containing 0.2 mg/ml of the voltage-sensitive merocyanine-rhodanine dye NK2761 (Hayashibara Biochemical Laboratories/Kankoh-Shikiso Kenkyusho, Okayama, Japan, 27–28). The excess (unbound) dye was washed away with dye-free Tyrode's solution before recording. The dye NK2761 has been used previously to record transmembrane potentials in the embryonic chick spinal cord [21]. In one experiment another dye (NK3041) was used, and the results were similar to those with NK2761.

### Electrical stimulation and recording

The cut ends of the dorsal and ventral roots were drawn into suction electrodes separately for recording and/or stimulation. Ventral root signals were amplified 1,000×and filtered from DC to 1 or 3 kHz. Evoked episodes of rhythmic activity were initiated by stimulating a dorsal root with a positive (depolarizing) square current pulse (10–20 µA/300 µsec). The motor nucleus was identified optically by stimulating a ventral root with an antidromic train (20Hz, 0.5sec, 40–50 µA/300 µsec) and recording the location of the evoked optical signals.

### Optical recording

An array of 124 photodiodes was used for the optical recording and 4 additional channels were used to record the electrical activity from the ventral roots on each side of the cord (Wutech, Red Shirt Imaging). The tissue was trans-illuminated with a 100 W tungsten-halogen lamp and a 10×or a 20×water immersion objective (NA 0.3 and 0.4 respectively) which projected the rostral surface of the slice onto the array. Unless otherwise indicated the tissue was illuminated at 710 nm. The photocurrent from each photodetector was individually amplified through a two-stage amplifier system. The first stage of amplification performed a current-to-voltage conversion using a feedback resistor of 20 MΩ. The signals were then amplified and filtered by a second stage amplifier before digitizing. This second stage provided a voltage gain of 1,000. This parallel amplifier arrangement [29] allows a low dark noise (10^−6^ of the illumination intensity), a large dynamic range (17–21 bits), and a fast sampling rate. The signals were high-pass filtered with a 5 sec time constant to provide a pseudo DC recording, and low-pass filtered using a four-pole Bessel analog with a 300 Hz corner frequency. The sensitivity of optical recording using dye absorption signals is comparable to that of local field potential recordings and absorption dyes have much lower phototoxicity than fluorescent voltage sensitive dyes [30]. The optical data were analyzed using the program NeuroPlex (A. Cohen and C. Falk; RedShirtImaging, LLC.) or custom routines written in Matlab (The Mathworks).

To establish the position of the array over the spinal cord the outline of the array was projected onto a piece of translucent tape over one of the camera ports. The image of the slice was positioned within this outline and the combined image photographed.

### Quantification of Optical Signals

We measured the timing of optical activity during each cycle after first bandpass filtering the digitized signals. For VSD signals, the first cycle was filtered between 0.1–50 Hz and subsequent cycles between 0.1–10 or 20Hz. We used the wider bandwidth for the first cycle because its rise time was faster than subsequent cycles. The slower intrinsic signals were bandpass filtered at 0.1–5 or 10Hz. We compared the timing of the electrical activity recorded from the ventral roots with the optical signals averaged from diodes over three different regions of the cord: The ventral area or the motoneuronal region as identified by antidromic stimulation; an intermediate region and over the dorsal horn. Typically the signals from 4–6 adjacent diodes in each region were averaged together. We then identified the individual cycles based on the onset of discharge of the integrated (25–100 ms) and rectified neurogram from the ventral root discharge on the same side of the cord as the diodes. The neural and optical signals were normalized and the onset of the activity defined as the time at which the signal crossed a level corresponding to 10% of the peak of the normalized signal. This time was referenced to the start of the slow potential recorded from the ipsilateral ventral root.

### Labeling of motoneurons and dorsal root sensory afferents

At the end of the experiment, nine cord slices were immersion fixed in 4% paraformaldehyde overnight. The following day, the slices were pinned to the base of a Petri dish coated with Sylgard. The ventral and dorsal roots that had been used in the optical experiment were identified and crystals of DiI and DiO (Molecular Probes) were carefully placed at the edge of the roots (6 cords with both dorsal and ventral roots labeled; 3 cords with just ventral roots labeled). Anterograde labeling of sensory afferents and retrograde labeling of motoneurons was allowed to occur for approximately 5–7 days, while the slices were kept in 4% paraformaldehyde at 40°C. After this period, the slices were immersed in melted 5% Agar dissolved in PBS and then cut serially in transverse sections (thickness 70 µm) using a Vibratome. The sections were collected on slides, coversliped and examined with a confocal microscope (LSM 510, Zeiss) equipped with two visible lasers (excitation wavelengths 488 and 543 nm for green (DiI) and red (DiO) respectively) and their corresponding filters (emission filter bandwidth for FITC: 500–530 nm and for Rhodamine: 560–615 nm). Sections were scanned at 1 µm intervals in the z-axis using multi-tracking to avoid cross excitation of the two dyes.

### Definition of Terms for Episodes of Activity

**Figure 1:**
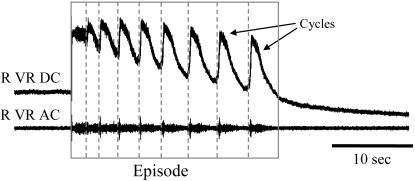
Structure of an episode of activity. Electrical activity recorded from a ventral root during a single episode of rhythmic activity. The duration of the complete episode is demarcated by the grey box. The onset of the individual cycles is defined by the dotted grey lines. 2 cycles are identified at the end of the episode. Data from an E8 embryo. R VR DC-Right Ventral Root DC recording; R VR AC-Right Ventral Root AC recording high pass filtered to show the discharge pattern.

Episodes of activity occur spontaneously or can be evoked by a single stimulus to the dorsal or ventral roots. The inter-episode interval is defined as the time from the end of a spontaneous episode to the start of the next. A typical episode is illustrated in figure 1, showing the individual cycles and identifying their time of onset.

## Results

In the first part of the paper, we describe our ability to optically resolve both threshold and subthreshold neuronal activity evoked by antidromic stimulation of motoneurons. We then show that both VSD and intrinsic optical signals accompany episodes of rhythmic activity. In the last part of the paper, we use the optical methods to identify the spatial source of activity at the beginning of each cycle of activity in an episode.

### Antidromic Stimulation of Motoneurons

**Figure 2:**
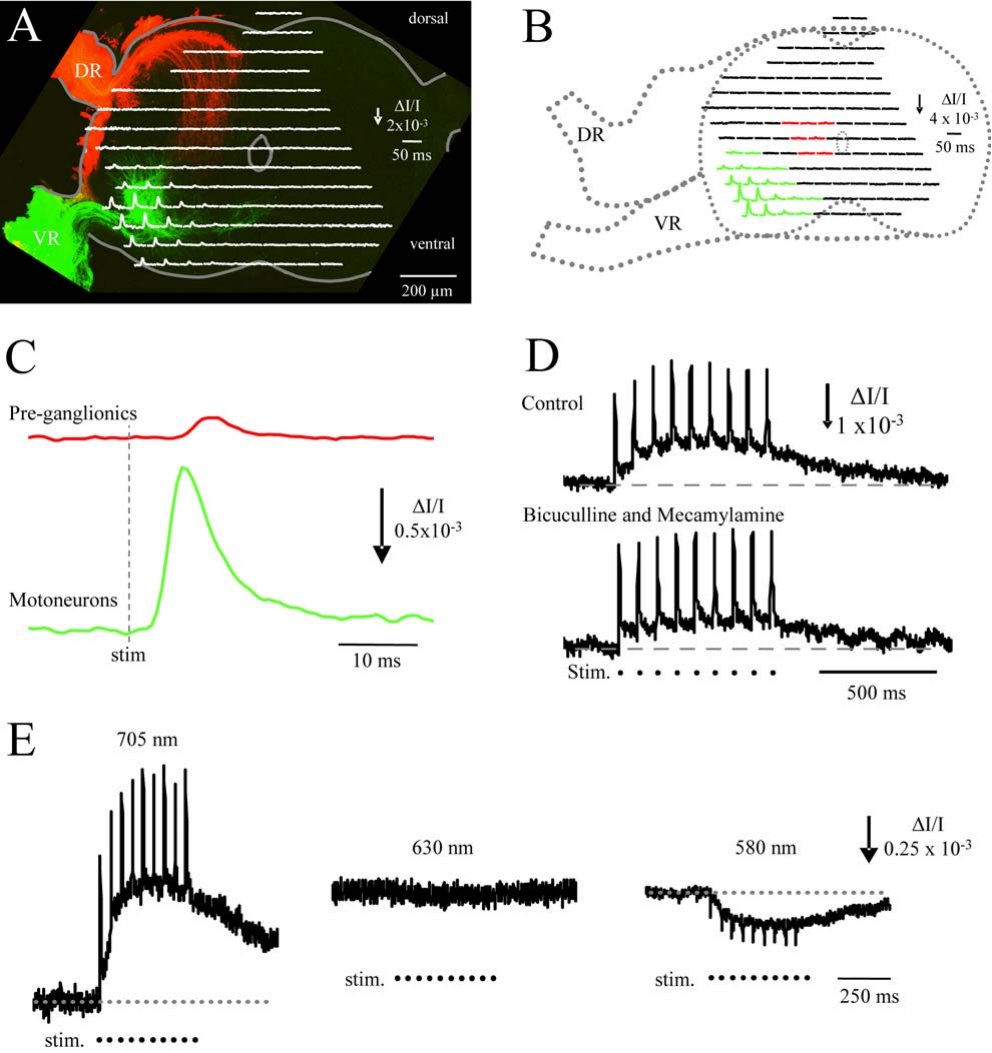
Antidromic stimulation of the ventral roots to identify the location of the motor nucleus. A. The optical responses recorded from the photodiode array are shown superimposed over a confocal transverse section of the slice in which the recordings were made. After the optical experiment, the slice was fixed and motoneurons were labeled retrogradely with DiI (green) applied to the ventral root and primary afferents were labeled anterogradely with DiO (red) applied to the dorsal root. Each optical signal is the response to a single antidromic stimulus averaged from a train of stimuli (8 stimuli at 20 Hz) and recorded in the presence of a cholinergic (mecamylamine 50 µM) and a GABAA (bicuculline 50 µM) antagonist to block the synaptic inputs from R-interneurons onto motoneurons (33). The largest antidromic responses coincided with the location of motoneuron cell bodies. B. Antidromically-evoked optical responses from another cord in which it was possible to detect antidromic spikes adjacent to the central canal at a location corresponding to that of preganglionic neurons (red traces), in addition to those over the motor nucleus (green traces). DR-dorsal root; VR-Ventral Root. The colored traces in panel B have been averaged and are displayed on an expanded scale in C. It can be seen that the antidromic responses near the central canal are smaller and have a longer latency that those recorded from the motor nucleus. D. The slow antidromic responses recorded over motoneurons are depressed in the presence of the nicotinic cholinergic antagonist mecamylamine and the GABAA receptor antagonist bicuculline to block the recurrent synaptic input from R-interneurons to motoneurons. The upper trace (control) was obtained under control conditions and the lower trace (bicuculline and mecamylamine) in the presence of the drugs. The time of the ventral root stimuli (stim.) are marked by the dots beneath the traces. E. Spectrum of optical responses recorded over motoneurons and evoked by ventral root stimulation under control conditions. The signals were obtained at three different illumination wavelengths in response to a train of ventral root stimuli (stim.). Note the reversal of the signals at 580 nm and the absence of a significant signal at 630 nm, the isosbestic point of the dye. The times of the ventral root stimuli (stim.) are indicated beneath the traces. In this, and all other figures, the vertical calibration arrows indicate the direction of increased light transmission (decreased light absorption). Data in A from an E11 embryo and in B, C and D from an E10 embryo. Data in E from an E11 embryo.

To establish the ability of our optical system to detect neural activity we measured VSD signals over motoneurons during antidromic stimulation of the ventral root. Figure 2A shows the optical responses to a single stimulus-averaged from 8 successive stimuli applied to the ventral root (8 stimuli at 20Hz)-superimposed over a confocal section of the fixed cord in which the motoneurons were retrogradely labelled with DiI (green) and the primary afferents anterogradely labeled with DiO (red). The optical response comprised a transient *decrease* in light intensity (upward signal) recorded from the ventrolateral part of the cord where motoneurons and their dendrites are located. The antidromic signals were largest over the cell bodies but they could also be detected over motoneuron dendrites. Dendritic signals were generally smaller than the somatic signals possibly because the dendrites were not completely invaded by the antidromic action potential.

The amplitude and latency of the optical antidromic spikes were measured in responses averaged from all of the diodes exhibiting an antidromic response (green traces in fig. 2B and C). The amplitude of the fractional decrease in light intensity (ΔI/I) during the antidromic spike was 1.0 (±0.2)×10^−3^ and its average latency from the stimulus was 2.6 ms (n = 5 exp). This latency is similar to that of antidromic spikes recorded intracellularly from motoneurons [26]. In most experiments, the antidromically-evoked optical spikes were restricted to the ventrolateral part of the cord. However, as illustrated in fig 2B and C, it was also possible to detect VSD spikes near the central canal ipsilateral to the stimulus (fig. 2B and C) in a region corresponding to the location of autonomic pre-ganglionic neurons [31]–[32]. The amplitude of these optical spikes was smaller than those recorded over the motor nucleus and their latency was longer.

We then investigated the pharmacology of the antidromic responses to a stimulus train delivered to the ventral root at 20Hz. Figure 1D (control) shows that the optical responses evoked by an antidromic train delivered to the ventral root (dorsal roots cut) consisted of a slow response on which spikes were superimposed and which were recorded over the region containing motoneurons and their dendrites. The slow responses were always recorded together with spikes suggesting that they originated from motoneurons. We hypothesized that the slow component of the antidromically-evoked signal was due to the recurrent synaptic activation of motoneurons by R-interneurons [18], [33]. R-interneurons project primarily GABAergic synapses onto motoneurons and are activated by the recurrent cholinergic/glutamatergic collaterals of motoneurons. Therefore, to establish if the slow component originated from this source, we compared the antidromic signals before and after bath-application of the cholinergic antagonist mecamylamine (50 µM) and the GABA_A_ antagonist bicuculline (50 µM) to block these recurrent synaptic potentials in motoneurons. Under control conditions, the averaged fractional decrease in light transmission (ΔI/I) of the antidromically-evoked slow signal was 0.8×10^−3^±0.2 and this was reduced by 49% to 0.4×10^−3^±0.1 (mean±SD; n = 5 experiments) in the presence of bicuculline and mecamylamine (fig. 2D, lowest panel). This difference was statistically significant (p<0.008). In two additional experiments, bath-application of mecamylamine and the muscarinic cholinergic antagonist atropine reduced the slow signal amplitude by 70% from 1.9×10^−3^ (control) to 0.6×10^−3^. The signal recovered partially during washout (to 1.1×10^−3^ or 55% of control).

To ensure that the antidromic signals were derived from the dye and were not due to changes in the light transmission of the tissue, we examined the synaptic responses and their spectrum during a train of stimuli applied to the ventral roots. As shown in fig. 2E, both the spike and the slow components of the response reversed at 580 nm and were largely abolished at 630 nm-the isosbestic point of the dye [34]. This spectral response indicates that both the slow depolarizing signal and the spikes are VSD signals and are not contaminated by light scattering (intrinsic optical signals). We did find, however, that longer antidromic stimulus trains (2 sec) were accompanied by delayed intrinsic optical signals (data not shown).

Collectively, these results demonstrate that the imaging technique we are using has the temporal resolution and sensitivity to detect synchronized fast antidromic spikes and the slow subthreshold synaptic potentials generated in motoneurons by the recurrent activation of R-interneurons.

### Intrinsic and VSD signals accompanying episodes of rhythmic activity

**Figure 3:**
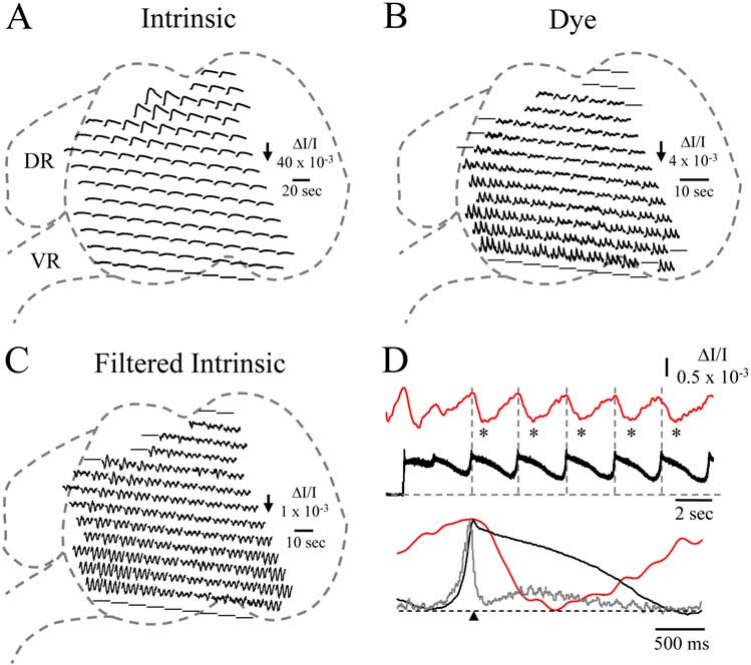
Comparison of the spatial distribution of two kinds of intrinsic signal and dye-related signals recorded from the cut, transverse face of the spinal cord. A–C. Distribution of intrinsic (A), dye-related (B) and filtered intrinsic (C) signals during an episode of rhythmic activity. The signals in A and C were obtained in an unstained slice which was subsequently stained with the voltage sensitive dye NK2671 to provide the VSD signals shown in B. The data were acquired at an interval of 0.42 ms. The signals in A were unfiltered and those in B were low pass filtered at 100Hz. The signals shown in D were obtained by bandpass (0.1–10 Hz) filtering the signals in A. Data in A is from a complete episode, while the signals shown in B and C were from 3 cycles from within the episode. Note that the dye signals and the filtered intrinsic signals exhibit a similar distribution of rhythmicity with the highest amplitude in the ventrolateral part of the cord. DR-dorsal root; VR-ventral root. D. The upper two traces compare the time course of the slow electrical activity (black trace) with the intrinsic optical signal recorded over the lateral motor column (red trace). Note that the peak of the optical signal is delayed with respect to the peak of the slow electrical signal. The lower traces are the cycle-averaged electrical (grey-integrated neurogram; black-low pass filtered slow potential) and optical (red-lateral motor column optical signal) responses generated from the cycles identified by the asterisks. The average was triggered from the peak of the slow potential as indicated by the vertical dotted grey lines. Data obtained from an E11 embryo.

In the next set of experiments, we examined the optical signals accompanying episodes of rhythmic activity. Previous work has shown that optical signals recorded from the isolated spinal cord include both VSD and intrinsic components [34]–[37]. To establish the nature of the intrinsic signals accompanying episodes of rhythmic activity, we initially documented the activity-dependent optical signals generated in unstained preparations. We found that large optical signals could be recorded during rhythmic activity. The intrinsic signal comprised two components each of which varied systematically across the transverse plane of the slice. The dominant component was a slow *decrease* in light transmission (upward signal in fig. 3A) that peaked within the first two or three cycles of activity and then decayed for the remainder of the episode. The peak amplitude of this signal was greatest in the dorsal area (ΔI/I: 16.1±5.6×10^−3^, n = 8; E10-11 embryos) and became progressively smaller in the intermediate region (ΔI/I: 3.5±0.9×10^−3^, n = 7 exp) to reach a minimum over the motoneurons (ΔI/I: 3±1.2×10^−3^, n = 8 exp).

The differences in the amplitudes of the ventral vs. dorsal and the intermediate vs. dorsal signals were significant (p<.005). Superimposed on this slow signal was a fast component exhibiting a rhythmic *increase* in light transmission (downward signal in fig. 3C and D) associated with each cycle of activity. This fast signal was isolated from the slow component by band-pass (0.1–5 or 10Hz) filtering. The rhythmic optical signal correlated well with the electrical activity recorded from the ventral root although its peak was delayed compared to the peak electrical activity (fig. 3D).

The fast component had a maximum peak to peak amplitude over the lateral motor column (ΔI/I: 0.46±0.3×10^−3^, n = 7 exp) declining in the intermediate region (ΔI/I: 0.27±0.2×10^−3^, n = 7 exp) to reach a minimum over the dorsal part of the cord (ΔI/I: 0.13±0.1×10^−3^, n = 7 exp; The difference between the ventral and dorsal amplitudes was statistically significant at p<0.05 but the difference between the ventral and intermediate amplitudes was not). This spatial distribution in the amplitude of the fast intrinsic signal was similar to that of the VSD signals accompanying each cycle of rhythmic discharge (fig. 3B) suggesting that this component of the intrinsic signal is generated by the rhythmic synaptic and/or spike activity of the motoneurons. Both the VSD and the fast intrinsic signals were widely synchronized across the transverse face of the cord.

### Optical signals accompanying the onset of spontaneous episodes

In the next set of experiments, we examined the optical signals accompanying the onset of *spontaneous* episodes. We wanted to confirm our previous observations (obtained using calcium imaging) that earliest optical activity at the *onset* of spontaneous episodes originated from the ventrolateral part of the cord [16]. This was necessary because the earlier experiments used relatively slow video rate imaging (30 frames/sec) which detects spike activity rather than subthreshold potentials. As a result, subthreshold activity outside the motor nucleus might have been missed. In the present work, the temporal resolution was much higher (∼500–1800 frames/sec) than in the previous work, and as we discussed earlier (see fig. 2D and E), it was also possible to detect subthreshold synaptic activity.

**Figure 4:**
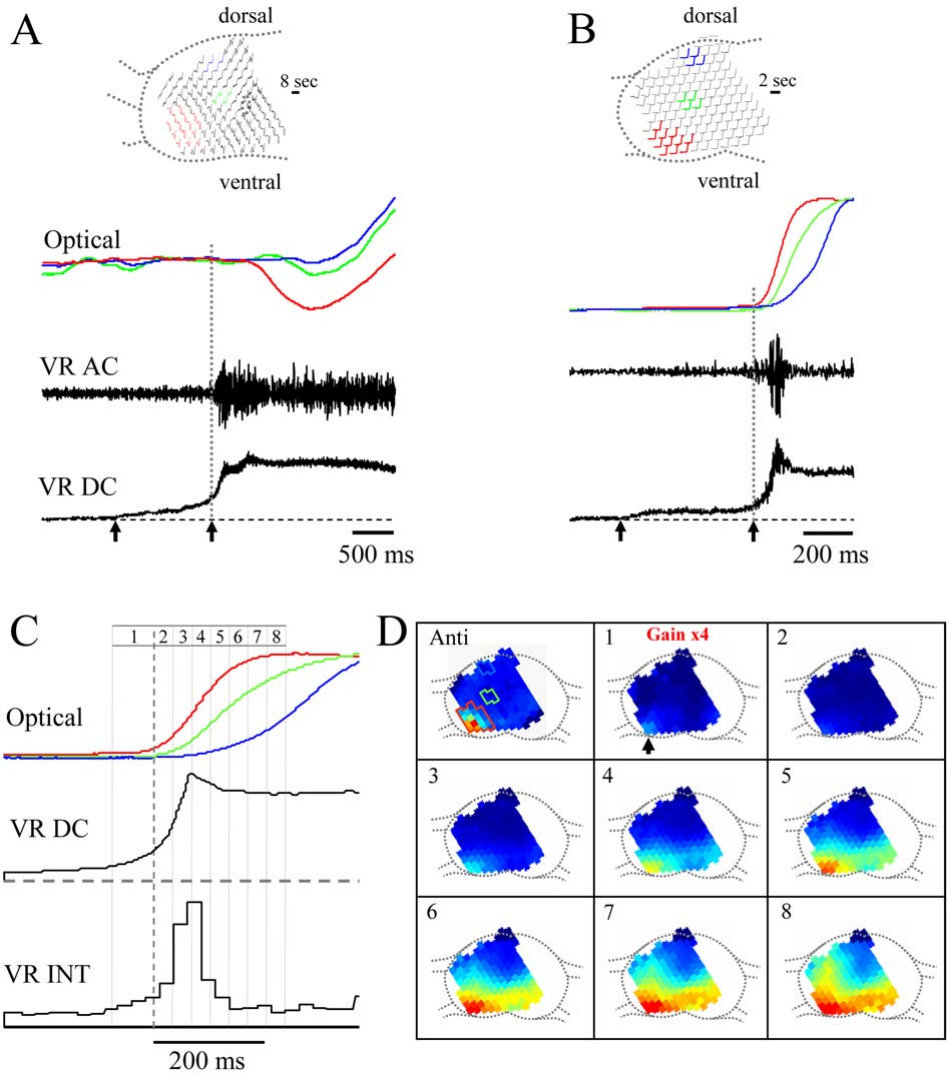
Optical signals originate ventrolaterally at the onset of a spontaneous episode. Both intrinsic (A) and dye-related (B) optical signals originate over the motor column at the onset of a spontaneous episode. Each optical signal was spatially averaged from the diodes shown in the schematic of the cord above the traces and then normalized to its peak amplitude. The electrical activity was simultaneously recorded from the ipsilateral ventral root (VR). DC-unfiltered. AC-band pass filtered from 20-312Hz. The arrows delineate the initial slow rise of the depolarization and the onset of discharge (dotted gray line) in the ventral root. Data in A were obtained from an E9 embryo, while those in B were from an E10 embryo. C. Spread of dye-related activity at the beginning of a spontaneously occurring episode. The data were averaged from two E10 embryos and synchronized to the onset of the ventral root discharge (dotted gray line). The smoothed DC ventral root potential (VR DC) and the integrated ventral root discharge (VR INT-25ms integration time) are displayed together with the optical responses from three different cord regions (ventral/lateral motor column-red; intermediate-green; dorsal-blue). Each record was averaged from several adjacent diodes (shown in panel A of the montage illustrated in D) and normalized to its peak amplitude. Data were obtained at a sampling interval of 0.64ms. The gray lines over the traces in C, delineate the frames that were averaged to produce the pseudocolored montage. D. Montage of the pseudocolored diode array signals superimposed on the outline of the transverse face of the cord slice. The number in each panel corresponds to the numbered intervals over the electrical and optical traces. The first image (Anti) in the sequence is a pseudocolored image of the optical signals during antidromic stimulation of the ipsilateral ventral root to identify the location of the lateral motor column. The colored regions on the image identify the location of the diodes whose signals were averaged to produce the optical records in C. The second image (1, Gain×4) was obtained before the onset of discharge (vertical dotted gray line in C) and was averaged from 105 frames (67ms/frame) is displayed at 4×the gain of the remaining images. All remaining images (2–8) were averaged from 45 frames (29ms/frame). The arrow shows the earliest detectable activity occurs over the lateral motor column.

Figure 4 shows the optical signals in unstained (fig. 4A) and dye-stained (fig. 4B) slices recorded at the beginning of a spontaneous episode. As shown in the lower panels of fig. 4A and B, the ventral root electrical signal depolarizes in two phases at episode onset-an initial slow rise that is accompanied by a low level of discharge (demarcated by arrows in the lowest panels of figs 4A and B) followed by an abrupt increase that is associated with the onset of intense firing and the onset of the episode proper (second arrow, figs. 4A and B; see also refs 17,18). We found that both the intrinsic and the VSD signals began first over the lateral motor column. To quantify the timing of the *intrinsic* optical signals we measured their onset with respect to the onset of the first detectable depolarization recorded from the ipsilateral ventral root (first arrow figs. 4A and B). In 3 experiments, the onset of the ventral intrinsic optical signal began 1140±643 ms *after* the initial ventral root depolarization. These measurements lengthened in the ventrodorsal direction to 1493±833ms for the intermediate region and 1975±1279 ms for the dorsal region.

A similar ventrodorsal sequence was observed for the VSD signals (fig. 4B). At episode onset, optical activity in the ventral area began 97±83 ms *before* the onset of the ventral root depolarization lengthening to 168±169 ms and 384±269 ms in the intermediate and dorsal regions respectively (n = 4 experiments, both the intermediate and dorsal latencies were significantly different from the ventral latency; p<0.05). When the VSD signals were made contralateral to the initial ventral root activity, the onset latencies were longer but they exhibited the same ventrodorsal sequence. Ventral: 287±93 ms; Intermediate 450±249 ms; Dorsal 465±180 ms (n = 3 exp).

To visualize this pattern of propagation in more detail, we constructed pseudocolored maps of the VSD signals over the different diodes at the beginning of a spontaneous episode. In 4/7 experiments the electrical activity began on the same side of the cord as the diode array as determined by the ventral root electrical recordings. In the remaining three, the ventral root activity began contralateral to the array. To visualize the small signals accompanying the slow ventral root depolarization at episode onset, we averaged the data from two of the four experiments in which electrical activity began on the same side as the diodes (fig. 4C and D). We first identified the location of motoneurons by antidromically stimulating the ventral root (fig. 4D, first panel Anti). This allowed us to establish that the earliest optical signal occurred over the lateral motor column. This activity progressively intensified and spread medially to the contralateral side of the cord (panels 4D–4 and 5) from where it spread dorsally (panels 4D-6 to 8). The optical signal rose rapidly, coincident with the onset of the discharge recorded from the ipsilateral ventral root. These findings are consistent with the idea that cells within the lateral motor column are the first to become active at the beginning of a spontaneous episode.

### Spatiotemporal organization of activity in later cycles

In the next set of experiments, we examined the timing of activity in the subsequent cycles of the episode. As before, we examined the optical activity in 3 different regions and measured its timing with respect to the onset of the electrical activity recorded from the ventral roots. Episodes were triggered by a single stimulus to the ipsilateral dorsal root.

**Figure 5:**
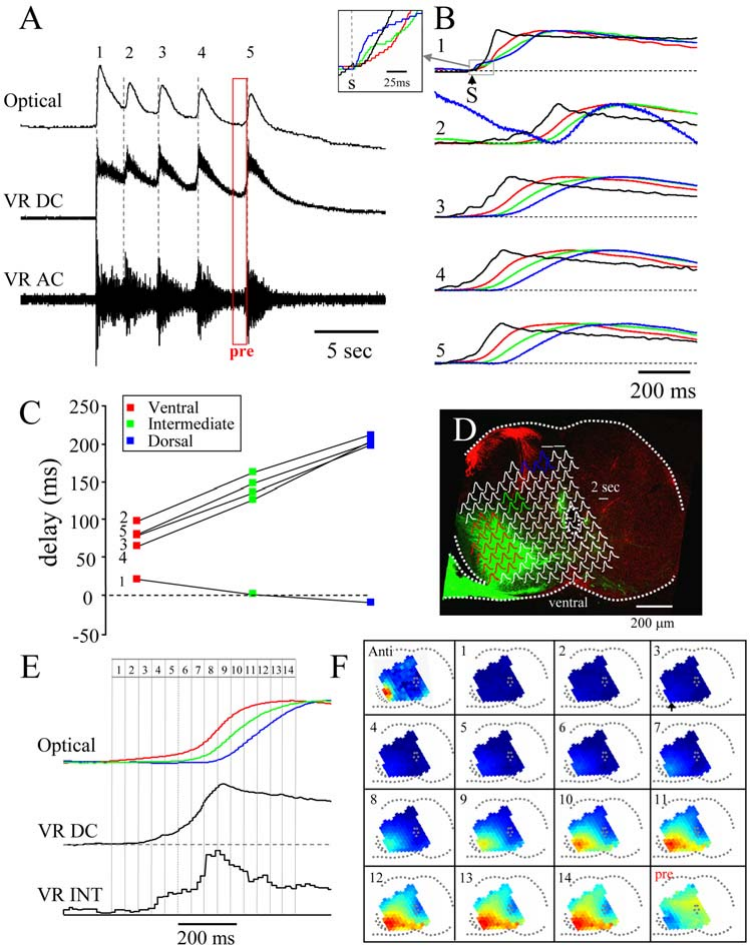
Timing of voltage-sensitive dye signals recorded from different regions of the cord at the onset of individual cycles. A. Electrical and optical recordings from an E10 embryo during an episode initiated by a dorsal root stimulus. The optical recording is from a single diode over the later motor column ipsilateral to the neural recordings. The dotted grey lines demarcate the onset of discharge in each cycle of activity. B. Electrical (black) and optical recordings from 3 different regions of the cord. As in previous figures, the red traces were averaged from diodes over the lateral motor column, the green traces from over the intermediate region and the blue traces from over the dorsal region. The diodes and their locations are shown in panel D. The neurogram is the DC trace low pass filtered between DC-20Hz. The episode was triggered by a single stimulus to the ipsilateral dorsal root at the time marked by the arrow (S). The numbered cycles correspond to those in panel A. The initial part of the first cycle (demarcated by a gray box) has been blown up in the inset to show the timing of the earliest optical and electrical activity. C. Quantification of the timing of optical activity in each cycle with respect to the onset of the electrical activity recorded from the ventral root. The onset delay of the optical signals with respect to the onset of the electrical signal is plotted for each cycle for each of the three regions. The numbers beside the plots correspond to the numbered cycles shown in A and B. Note that the dorsal optical activity precedes that of the electrical activity in the first cycle. Moreover the first cycle exhibits a dorsoventral sequence of activation but all of the other cycles are activated ventrodorsally. D. Location of the diodes whose signals were averaged to produce the traces shown in panels B and E. The diode signals are superimposed over a stained section in which the motoneurons were labeled with DiO and the dorsal root afferents were labeled with DiI. E. Cycle-triggered, averaged DC ventral root potential (VR DC-smoothed with a 20 point moving average) and integrated ventral root discharge (VR INT) together with the optical responses from the three different cord regions ipsilateral to the earliest ventral root activity. The last three cycles (3–5 in A) were averaged for these records. As before, each trace was averaged from several adjacent diodes (shown in D) and was normalized to its peak amplitude. Data were obtained at a sampling interval of 1.011 ms. F. Montage showing the pseudocolored diode array signals superimposed on the outline of the cord slice. Each image is the average of 45 frames obtained at the times indicated by the numbered regions over the traces in E. The first panel in the sequence (Anti) is an image generated during antidromic stimulation of the ventral root to identify the location of the lateral motor column. The arrow in image 3 shows the initial activity within the lateral motor column. The last image in the series (pre) was averaged from the frames delimited by the red rectangle marked pre in panel A. This image shows the spatial distribution of activity just before the last cycle.

**Figure 6:**
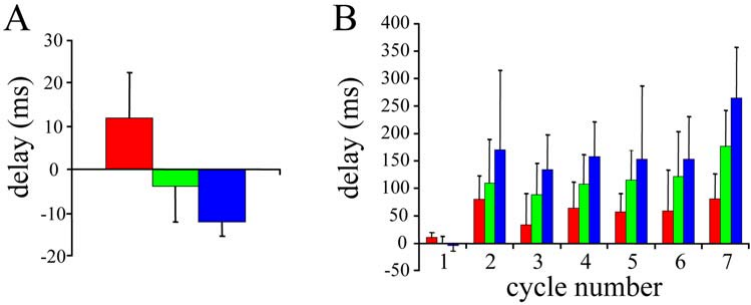
Summary of the timing of activity in different cycles within episodes evoked by a stimulus to the dorsal root. A. Sequence of activity for the first cycle in 5 experiments in which the dorsal optical activity preceded the onset of the electrical activity. B. Average delays of the optical signals measured from the onset of the ipsilateral electrical activity for 7 experiments. Time 0 corresponds to the onset of slow electrical activity recorded from the ipsilateral ventral root. The color code is the same as in the previous figures. Data are mean±SEM.

Following stimulation of the dorsal root, we observed a *dorsoventral* sequence of activation during the first cycle and *ventrodorsal* sequence of activation in all of the subsequent cycles (fig. 5 and 6). In the example illustrated in fig. 5, optical activity began in the dorsal part of the cord 8ms before the onset of the slow potential recorded from the ipsilateral ventral root (see inset fig. 5B). The activity propagated ventrally reaching the intermediate diodes 11 ms later and the ventral diodes 31ms later. This sequence of activation is much faster than that occurring at the onset of a spontaneous episode presumably because the afferent stimulus provides a synchronized transient. The dorsoventral propagation of activity following a dorsal root stimulus is consistent with previous work in the chick [21] and the neonatal rat [38]. By contrast, in all of the remaining cycles the ventral activity began first. In the example shown in fig. 5, the distance between the center of the averaged dorsal and ventral diodes was∼525 µm (see fig. 5D) which gives a dorsoventral propagation velocity of 525/31 or 17 µm/ms.

The average delay from the slow ventral root potential onset for the remaining cycles was 81ms for the ventral diodes, 143 ms for the intermediate diodes and 204 for the dorsal diodes. Thus, for these cycles, direction of propagation was reversed and its velocity was significantly slower (525/123 or 4.3 µm/ms) than the initial dorsoventral activity. In addition, we found that the peak motoneuronal activity recorded optically (red traces, fig. 5B) was delayed with respect to the peak ventral root activity (black traces fig. 5B) except for the first cycle of the episode. The delay probably arises for two reasons. First, the electrical activity recorded from the ventral root comprises discharge superimposed on a slow population synaptic potential. When this signal is low pass filtered (as in fig. 5) the peak discharge distorts and advances the peak of the slow potential. Second, the VSD signals are superimposed on intrinsic optical signals that cannot be completely eliminated by bandpass filtering. In particular, as we have discussed earlier, each cycle of activity is accompanied by a rhythmic *increase* in light transmission (a downward signal) that peaks later than the electrical activity (see fig. 3) and which may delay the peak of the VSD signal.

The spread of activity was visualized by creating montages of the pseudocolored optical signals as illustrated in fig. 5F. To maximize the signal to noise ratio, we averaged the last three cycles of the episode illustrated in fig. 5A and normalized them to their peak amplitude. The earliest activity began within and around the lateral motor column, and spread diffusely throughout the ventrolateral region. Coincident with the onset of the peak discharge recorded from the ventral root, the optical activity intensified within the lateral motor column, spreading both dorsally and medially.

In 7 experiments we quantified the timing of activity for each cycle in which an episode of activity was initiated by an ipsilateral stimulus to the dorsal root. In 5 of these experiments, including the experiment shown in fig 5, the dorsal activity in the first cycle began before the onset of the ventral root slow potential −3.6±5.3 ms (mean±S.E.M). The activity propagated dorsoventrally to reach the intermediate region 1±4.2 ms and the lateral motor column 11.1±3 ms after the ventral root slow potential onset (see fig. 6A). We then quantified the average delays for the optical activity in all the later cycles, for the three different regions (ventral, intermediate, dorsal) in 7 experiments (including the 5 above) as shown in fig. 6B. As for the individual experiment of figure 5, the average delay lengthened ventrodorsally in each of the remaining cycles.

## Discussion

In this paper we have investigated the spatial origin of activity during each cycle of episodic activity. We show that the earliest detectable optical activity at cycle onset originates in the ventrolateral part of the cord where motoneurons are located. We found that both intrinsic and VSD signals propagate in the ventromedial and ventrodorsal direction at the onset of each cycle of an episode. Collectively, these results are consistent with the idea that motoneuron activity is critical for the generation of rhythmic activity and that the synaptic connections of motoneurons and their intraspinal targets are likely sites for the fast synaptic depression that has been hypothesized to underlie cycling. In addition, we have identified two types of intrinsic signal that accompany episodes of neural activity. One component is slow and up to 10 times the amplitude of the VSD signals. Superimposed on this signal is a much smaller rhythmic component that has a similar spatial distribution to the VSD signals.

### Antidromic Signals

Antidromic stimulation of the ventral roots in dye-stained cords resulted in a decrease in light transmission over motoneuron cell bodies and their dendrites. The antidromic signal comprised spikes superimposed on a slow signal. Both components reversed when the illumination was changed from 700 to 580 nm and they were almost abolished at 630 nm. This spectrum indicates that the signals were dye-related [34] and were not significantly contaminated by intrinsic changes in absorption. However, this conclusion only applied to short (∼1 sec) antidromic trains because longer trains were accompanied by delayed intrinsic signals.

We found that the slow component of the signal evoked by antidromic stimulation was depressed by cholinergic and GABAergic antagonists suggesting that it was mediated synaptically. The most likely source of this synaptic depolarization is the population of R-interneurons that are excited by cholinergic motoneuron collaterals and project GABA/glycinergic synapses back onto motoneurons [18], [33]. However, these antagonists did not completely block the slow component evoked by the antidromic stimulus train (fig. 2D). It is not clear what causes this residual slow signal. One possibility is that it results from the temporal summation of the after-depolarization accompanying each antidromic spike in motoneurons. The residual depolarization that persists in the presence of cholinergic antagonists alone, could reflect ventral root activation of R-interneurons by another transmitter system. Recently it has been shown that motoneuron recurrent collaterals release an excitatory amino acid as well as acetylcholine in the neonatal mouse [39]–[40]. In the chick embryo, this may also be the case, because stimulation of a ventral root evokes potentials in an adjacent ventral root and optical signals in R-interneurons that are depressed by glutamatergic antagonists [41]–[43].

### Two types of intrinsic optical signal accompany neural activity

In unstained slices, changes in light transmission accompanied spontaneous and evoked episodes of activity. During an episode, the intrinsic signal comprised two components. One was a slow *decrease* in light transmission that peaked within the first two or three cycles and decayed for the remainder of the episode. The peak amplitude of this component was largest in the dorsal horn and it became progressively smaller in the ventral direction. The second component, superimposed on the first, was a rhythmic *increase* in transmission that coincided with each cycle of depolarization and discharge recorded from the ventral root. The spatial distribution of this signal was similar to that of the VSD signal and the amplitude of both was maximal over the lateral motor column. These observations suggest that the fast intrinsic signal was produced by rhythmic neural activity.

In the brainstem of the chick embryo, local application of glutamate leads to an initial *increase* in light transmission followed by a decrease [36]. By contrast, local application of GABA or glycine leads to a *decrease* in transmitted light intensity [35] which might account for the observation that the largest decrease in light transmission occurred in the dorsal horn where GABAergic synaptic interactions are prominent [44]. Activity-dependent changes in light transmission have been attributed, in part, to corresponding alterations in cellular volume because local application of hypotonic or hypertonic solutions to the embryonic chick brainstem leads to an increase or decrease in light transmission respectively [36]. In the hippocampal slice, two types of intrinsic signal have been identified–associated with increased and decreased light transmission respectively. The decrease in light transmission (increased absorption) has been attributed to organelle swelling or dendritic beading but not to cell-volume changes. By contrast, increased light transmission has been identified with changes in cell volume [45]. An increase in light transmission has also been observed to accompany neurosecretion in the unstained mouse neurohypophysis. This signal was attributed to activity-dependent changes in light scattering at the active terminals [46]. It is possible that a component of the cyclical intrinsic signals we observed may also be due to this effect. Whatever the mechanism underlying intrinsic signals, it is clear that they can be used to monitor the spatiotemporal dynamics of activity within the spinal cord. As such, intrinsic optical signals can be used to complement the information obtained using calcium or voltage sensitive dye imaging, but without the risk of phototoxicity.

### Episode triggering and the spatiotemporal organization of activity within an episode

In the developing chick cord, an episode of activity is an all-or-none event that is maintained in the absence of the triggering event. Episodes can be triggered by non-specific stimuli including: a single stimulus applied to the dorsal roots or ventral roots or spontaneously occurring transient depolarizations [18]. Once triggered, the spatiotemporal dynamics of the episode become independent of the trigger. This highlights an important distinction between the triggering event and the spatial and temporal distribution of the activity within the episode once it has started. Since the triggering events are likely to be brief and small, they may not be detectable using current imaging methods. However, our VSD optical recordings show that once an episode is triggered, each cycle begins with optical activity in and around the lateral motor column.

These observations indicate that motoneurons and other neurons within the lateral motor column are the first to become recruited once the threshold for episode initiation has been reached. Our previous calcium imaging experiments have suggested that motoneuron excite the rest of the network via the activation of R-interneurons [18]. The synchronized recruitment of motoneurons in the absence of significant activity in other parts of the network strongly implies that motoneurons are connected by recurrent functionally excitatory synapses. We have shown previously that the reciprocal connections between R-interneurons and motoneurons are functionally excitatory and constitute a positive feedback loop [18]. Such positive-feedback connections will be enhanced by direct electrical and chemical connections between motoneurons such as those demonstrated to exist between mammalian motoneurons [39], [40].

In this regard, the motoneuronal/R-interneuronal network can be considered as a pacemaker driving activity in the rest of the network. However, as the more medial and dorsal parts of the network become active they will contribute synaptic excitation back onto motoneurons. It is reasonable to ask therefore whether the termination of a cycle depends on fast synaptic depression within the motoneuronal network and/or its connections to the remainder of the network or alternatively, to synaptic depression of the more dorsal and medial parts of the network that become active after motoneurons. Unfortunately, present techniques do not allow us to answer this question unequivocally. We note however, that optical maps taken just before the onset of a cycle (see fig. 5F, last panel marked pre) show a low level of activity within the motor nucleus in the presence of persistent activity outside the nucleus. This suggests that the activity of motoneurons has terminated before that of other parts of the network, consistent with a critical locus for the activity-dependent fast synaptic depression within this population of neurons.

When a cycle of motoneuron activity terminates, we hypothesize that the recurrent excitation in the remainder of the network is insufficient to maintain regenerative activity and it eventually dies out. The next cycle of motoneuron activity is presumably triggered by another transient event as the motoneuronal/R-interneuronal synapses recover from fast synaptic depression. The triggering event could arise within the motoneuronal/R-interneuron circuit itself or be derived from the decaying activity in dorsomedial parts of the network.

Although our results are consistent with the hypothesis that motoneuron activity is critical for rhythmicity, we cannot be certain that the optical signals recorded over the motor nucleus arise solely from motoneurons. This is because the optical signals may include components originating from the dendrites and axons of neurons outside the motor column, or from interneurons within or near the lateral motor column. Nonetheless, it seems likely that motoneurons will contribute the most membrane to this region, and hence will dominate the voltage dye dependent optical signal.

### Mechanisms of ventrodorsal activation

From the lateral motor column optical activity propagates ventrodorsally at ∼4 µm/ms, which is a similar rate to that reported earlier using calcium imaging [16]. This rate of propagation is too slow to be explained by axonal conduction delays. Indeed, the conduction velocity of motoneuron axons has been estimated to be ∼0.5 m/sec, or ∼100 times faster than the propagating activity. It seems more likely, therefore, that the activity spreads by the synaptic activation of neuronal groups coupled through relatively short-range synaptic connections. We propose that this activity originates in motoneurons because they are among the most mature neurons in the cord. Because of this, their synaptic connections with their interneuronal partners may be more developed than those of more dorsal interneurons consistent with the known ventrodorsal sequence of spinal cord maturation [47].
